# Complications in adolescent pregnancy: systematic review of the literature

**DOI:** 10.1590/S1679-45082015RW3127

**Published:** 2015

**Authors:** Walter Fernandes de Azevedo, Michele Baffi Diniz, Eduardo Sérgio Valério Borges da Fonseca, Lícia Maria Ricarte de Azevedo, Carla Braz Evangelista

**Affiliations:** 1 Universidade Federal da Paraíba, João Pessoa, PB, Brazil.; 2 Universidade Cruzeiro do Sul, São Paulo, SP, Brazil.

**Keywords:** Adolescent, Pregnancy, Pregnancy in adolescence, Pregnancy complications

## Abstract

Sexual activity during adolescence can lead to unwanted pregnancy, which in turn can result in serious maternal and fetal complications. The present study aimed to evaluate the complications related to adolescent pregnancy, through a systematic review using the Medical Subject Headings: “pregnancy complication” AND “adolescent” OR “pregnancy in adolescence”. Only full original articles in English or Portuguese with a clearly described methodology, were included. No qualitative studies, reviews or meta-analyses, editorials, case series, or case reports were included. The sample consisted of 15 articles; in that 10 were cross-sectional and 5 were cohort studies. The overall prevalence of adolescent pregnancy was 10%, and among the Brazilian studies, the adolescent pregnancy rate was 26%. The cesarean delivery rate was lower than that reported in the general population. The main maternal and neonatal complications were hypertensive disorders of pregnancy, prematurity and low birth weight, respectively. Adolescent pregnancy is related to increased frequency of neonatal and maternal complications and lower prevalence of cesarean delivery.

## INTRODUCTION

Sexual activity in adolescence initiates earlier and earlier, with immediate undesirable consequences, such as an increased frequency of sexually transmitted diseases (STD) and pregnancy, many times also undesired, which may therefore lead to an abortion.^(1)^

In Brazil, during the period from 2000 to 2006, the Live Birth Information System (SINASC, *Sistema de Informação sobre Nascidos Vivos*) recorded a decline in participation of births in mothers aged 15 to 19 years. However, the proportion of liveborns whose mothers were not in the age group under 14 years of age remained stable. In 2006, 51.4% of the liveborns were children of mothers aged up to 24 years, with approximately 1% of mothers in the age group under 14 years; 20.6% of the mothers aged from 15 to 19 years; and 29.9% of mothers aged between 20 and 24 years.^(2)^ In 2012, of the 2,905,789 liveborns, 560,147 (19.28%) were from adolescent mothers.^(3)^

From the biological point of view, among the consequences of pregnancy in adolescence are the high rates of hypertensive disorders of pregnancy, anemia, gestational diabetes, delivery complications, determining an increase in maternal and fetal mortality.^(4-6)^ It is important to note that some studies showed an increased trend of prenatal, intrapartum, and postpartum intercurrent events among pregnant adolescents.^(7,8)^

As to problems with the newborn, gestation during adolescence is associated with higher rates of low birth weight (LBW), preterm delivery, respiratory diseases, and birth trauma, besides a higher frequency of neonatal complications and infant mortality.^(9-11)^

Considering the high prevalence of adolescent gestation and its consequences, this study had the objective of analyzing complications related to adolescent pregnancy.

## METHODS

This is an a systematic literature review study that followed the recommendations proposed by Cochrane Collaboration.^(12,13)^

The guiding issue proposed for the study was: What are the complications related to adolescent pregnancies?

Data collection took place between May and August 2012, by means of an online search in the following databases, starting from the Virtual Health Library (VHL), MEDLINE (PubMed), Latin American and the Caribbean Health Sciences Information Literature (LILACS), and the Scientific Electronic Library Online (SciELO).

To find the articles, the following descriptors were used from the Medical Subject Headings (MeSH), of the PubMed/MEDLINE database: “pregnancy complication” AND “adolescent” OR “pregnancy in adolescence”.

Inclusion criteria included original articles, entirely available for free in the online version, in English and Portuguese, and during the period from 2002 to 2012. The study included randomized clinical trials, quasi-randomized clinical trials, observational analytical studies (case-control, prospective and retrospective cohort studies), and cross-sectional descriptive studies (on prevalence), which included a clear description of the methods used. Studies carried out with large samples of adults, but that included adolescents as a subgroup, were also included.

Not included were theoretical articles, investigations with an unclear description of methods used, manuscripts based on annual statistical reports (census data, and information obtained indirectly by means of graphs or archives), qualitative studies, reviews or meta-analyses, theses and dissertations, editorials, opinion articles, case series, care reports, studies with samples not representative of a population and prior to the year 2002.

The studies were initially stratified as per types of design, and posteriorly, as to the outcomes, following Cochrane’s methodology.^(14)^

The methodological quality of the systematic review was defined with the confidence that the design and report of the study were unbiased,^(15)^ and was evaluated independently by two reviewers in order to check if the inclusion and exclusion criteria had been met. In case of doubts or disagreement, a third reviewer was requested to issue an official opinion on whether or not the study should be included, according to Stocco.^(16)^ In the case of duplicate studies, the most recent one or that with the most complete information was included.

To evaluate methodological quality, and inclusion and exclusion criteria, the recommendations made by STROBE (The Strengthening the Reporting of Observational Studies in Epidemiology) were used.^(17,18)^ The assessment was divided into three study categories: (A) in cases of studies that satisfied a value of ≥80% of the criteria requested; (B) cases that satisfied 79 to 50% of the criteria; and (C) in cases that satisfied <50% of the criteria established.^(14,17,18)^ Thus, only the articles that reached a percentage >50% (classified as A or B) were considered of good quality and were included in the investigation.^(19)^

The data analyzed were synthetized and organized by means of figures, charts, and tables.

## RESULTS

**Figure 1:**
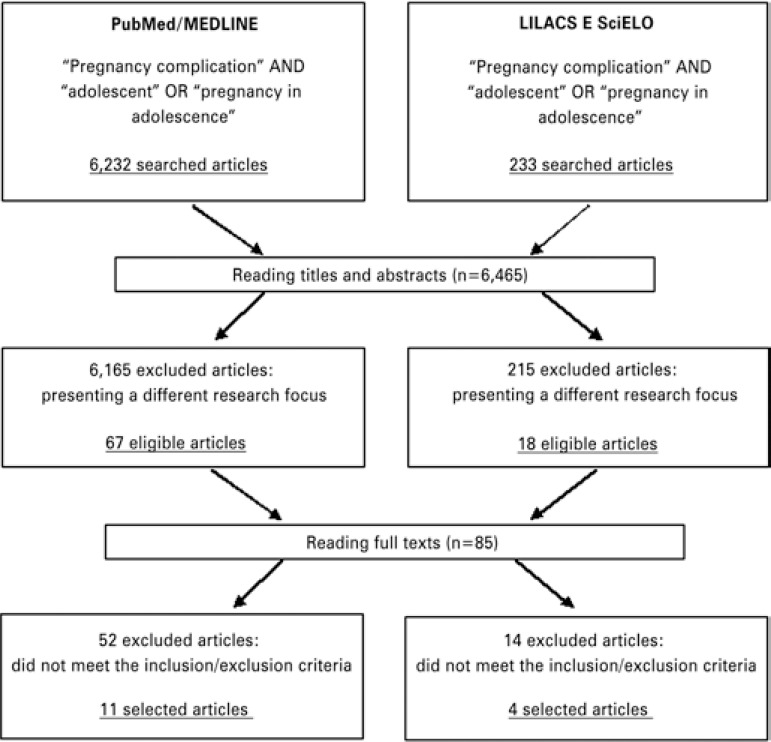
Flow chart for identification and selection of systematic review studies

The universe of the study was made up of 6,465 articles, 6,232 of them at PubMed/MEDLINE and 233 at LILACS and SciELO. After reading the titles and/or abstracts, 6,380 articles were excluded for presenting a focus different from the objective intended. Thus, of the 85 publications read entirely, 15 were selected that met the inclusion and exclusion criteria, as per figure 1.

Chart 1 shows the countries of origin, year of publication, and design of the studies included in the present review.

**Chart 1:** Relation of the studies included as per the countries of origin and the study design

Reference	Country	Study design	STROBE
Nili et al.^(20)^	Iran	Cross-sectional	B
Markovitz et al.^(21)^	United States	Retrospective cohort	B
Stewart et al.^(22)^	Nepal	Retrospective cohort	A
Kongnyuy et al.^(23)^	Cameroon	Prospective cohort	B
Reime et al.^(24)^	Germany	Retrospective cohort	A
Santos et al.^(25)^	Brazil	Cross-sectional	B
Muganyizi et al.^(26)^	Tanzania	Cross-sectional	B
Santos et al.^(27)^	Brazil	Cross-sectional	B
Khashan et al.^(28)^	England	Retrospective cohort	A
Mukhopadhyay et al.^(29)^	India	Cross-sectional	B
Oliveira et al.^(30)^	Brazil	Cross-sectional	B
Correia et al.^(31)^	Brazil	Cross-sectional	B
Lopoo^(32)^	USA	Cross-sectional	B
Martins et al.^(33)^	Brazil	Cross-sectional	B
Santos et al.^(34)^	Brazil	Cross-sectional	B

Of the 15 articles selected, 10 had a cross-sectional design and 5 were cohort, 4 of them retrospective and 1 prospective studies. As to year of publication, most of the studies were published during the period from 2008 to 2012. The studies were conducted predominantly in Brazil, United States, countries of Europe, Africa, and Asia. All the articles selected for systematic review showed STROBE percentages >50%, and 3 were classified as STROBE A and 12 as STROBE B.

Chart 2 shows the primary characteristics of the studies regarding pregnancy complications in adolescence.

**Chart 2:** Characteristics of the studies included regarding adolescent pregnancy complications (2002/2012)

Reference	Objective	Primary outcomes
Nili et al.^(20)^	To determine the frequency of pregnant adolescents and maternal and neonatal complications.	Maternal complications: pre-eclampsia, PRM, UTI, and heart and thyroid disease. Neonatal complications: prematurity, LBW, delayed intrauterine growth, and mortality.
Markovitz et al.^(21)^	To investigate the relation between infant mortality, socioeconomic level, and maternal age.	Child mortality, neonatal, and postnatal risks were significantly greater for younger adolescents.
Stewart et al.^(22)^	To determine the association between young maternal age and adverse outcomes.	Maternal age was associated with increased risk of prematurity among the primiparous.
Kongnyuy et al.^(23)^	To determine the adverse fetal complications associated with pregnancy in adolescence.	Fetal outcomes were LBW, prematurity and neonatal death. Maternal outcomes were eclampsia, pre-eclampsia, perineal laceration, and episiotomy.
Reime et al.^(24)^	To compare the risk of adverse outcomes in nulliparous adolescents and those who had an induced abortion or a previous delivery.	Adolescents with a prior delivery presented with greater perinatal, neonatal, and mortality risks. Those with a prior abortion presented with elevated risks for stillborn, prematurity, and VLBW.
Santos et al.^(25)^	To analyze the association between pregnancy in adolescence with LBW.	Among adolescents, LBW and prematurity were associated with low level of schooling, low number of prenatal visits, and late initiation of prenatal care.
Muganyizi et al.^(26)^	To establish the temporal changes in maternal age and its impacts for cesarean sections and LBW.	The proportion of adolescent mothers diminished progressively over time (1999-2005). The risk of cesarean sections increased and the risk of LBW was reduced.
Santos et al.^(27)^	To analyze the association between maternal age, perinatal results, and route of delivery.	Among the adolescents, a greater risk of prematurity, LBW, frequency of abortive method use, low number of prenatal visits, and late initiation of prenatal care were noted.
Khashan et al.^(28)^	To investigate the relation between the first and second pregnancies in adolescence and pre-term delivery, birth weight, and SGA.	The risk of premature birth increased in the first and second pregnancies. There was some evidence of a protective effect against SGA during the adolescent first gestation.
Mukhopadhyay et al.^(29)^	To compare the perinatal differences between adolescent and adult primigestas.	The adolescent mothers presented with greater proportion of premature deliveries, LBW, and stillborn.
Oliveira et al.^(30)^	To identify the effect of pregnancy on adolescence and risk factors for fetal and infant mortality.	Low weight and prematurity were determining factors of fetal and neonatal death. The risk of postnatal death was higher in the presence of multiparity, maternal morbidity, and LBW.
Correia et al.^(31)^	To investigate abortion in adolescents.	26.7% of the adolescents aborted primarily due to fear.
Lopoo^(32)^	To investigate the relation between adolescent pregnancy and complications of delivery.	The adolescents had lower rates of PROM, placenta previa, prolonged labor, breech presentation, among other complications.
Martins et al.^(33)^	To analyze the association between pregnancy in adolescence and prematurity.	There was an association between prematurity and the lower number of prenatal visits, late initiation of prenatal visits, LBW, and low level of schooling.
Santos et al.^(34)^	To identify the association between the maternal nutritional status and prenatal conditions with LBW and prematurity.	Birth weight was associated with the intergestational interval, pre-gestational weight, pre-gestational BMI, and total weight gain during gestation.

PRM: prolonged rupture of the membranes; UTI: urinary tract infection; LBW: low birth weight; VLBW: very low birth weight; SGA: small for gestational age; PROM: premature rupture of fetal membranes; BMI: body mass index.

In a study performed with 2,357 pregnant women, the frequency of adolescent women (aged under 18 years) was 4%. The most noted maternal complication was prolonged rupture of the membrane, with 20.3%, followed by pre-eclampsia (7.1%), thyroid diseases (7.1%), heart diseases (3%), and urinary tract infections (2%). Among the neonatal complications, the highlights were prematurity (39%), LBW (32%), and delayed intrauterine growth (12%). Neonatal mortality was described in 6.9% of the cases, and was significantly higher than the neonatal complications of the other deliveries.^(20)^

Another study performed with 265 adolescent mothers (aged ≤19 years) and with 832 mothers aged between 20 and 29 years showed that of the pregnancy-related maternal complications, the most frequent were eclampsia (OR=3.18), pre-eclampsia (OR=1.82), perineal tear (OR=1.45), and episiotomy (OR=1.82); while fetal complications were LBW (OR=1.71), prematurity (OR=1.77), and early neonatal death (OR=2.18).^(23)^

A study performed by means of analysis of data collected by the Texas of State Health Department between 1994 and 2003 assessed the complications that occurred during labor in 1,355,962 nulliparous mothers and showed that adolescent mothers (15 to 18 years) had lower rates intrapartum fever, excessive meconium, premature rupture of the membranes, placenta previa, prolonged labor, dysfunctional delivery, breech presentation, cephalopelvic disproportion, and umbilical cord prolapse, when compared to mothers aged between 25 and 29 years.^(32)^

Mukhopadhyay et al. compared the perinatal differences between 350 adolescents (13 to 19 years) and 350 adults (20 to 29 years), both groups of primigestas, by means of medical record analysis, and demonstrated that there was a greater proportion of premature deliveries (27.7%), LBW (38.95%), and rate of stillborns (5.1%), in comparison with adult mothers.^(29)^

One investigation researched the reasons that led the adolescent to provoke the abortion, relating the motivation with age and type of school they attended. Of the 2,592 adolescents that participated in the study, 182 (7.0%) referred having become pregnant and 149 (26.7%) having aborted. It was verified that the fear of the parent’s reaction (the most often cited reason), age, lack of support from the partner, and non-acceptance of the pregnancy were reasons that led the adolescents to provoke an abortion. The frequency of abortion was higher among the adolescents from public schools.^(31)^

A study conducted by means of data analysis of the residents of the State of Missouri (United States) during the years 1997 to 1999 investigated the relation between infant mortality (neonatal and postnatal), socioeconomic level, and maternal age. This study involved 10,131 adolescents between 12 and 17 years, 18,954 adolescents between 18 and 19 years, and 28,899 adults (20 to 34 years), and showed that the risks of infant (OR=1.95), neonatal (OR=1.69), and postnatal (OR=2.47) mortality were significantly greater among adolescents aged 12 to 17 years than among the adults (20 to 34 years). After adjustment for race, marital status, schooling level, smoking, prenatal care, and poverty, the risk of postnatal mortality (OR=1.73) remained significantly higher for younger adolescent mothers, but not the risk of neonatal mortality (OR=1.43).^(21)^

Researchers determined the association between a young maternal age and risk of LBW, small for gestational age (SGA), and premature delivery in 1,359 nulliparous (759) or uniparous (600) adolescents, who had diet supplementation with micronutrients and gave birth to a child analyzed within 72 hours after the delivery, from 1999 to 2001. The results showed that there was no difference in risk of LBW (OR=0.96) or SGA (OR=1.01) per year of maternal age increase among the primiparous mothers. Young maternal age did not affect the anthropometric data or gestational age of the offspring. Among primiparous adolescents, each year of increased maternal age was associated with an increase at birth in height, head and chest circumference, but not of the weight of the infant. Young maternal age (≤18 years) was associated with an increased risk of premature delivery among the primiparous mothers (OR=2.07).^(22)^

One study analyzed perinatal data gathered between 1990 and 1999, compared the risk of adverse outcomes in nulliparous adolescents (7,845) and adolescents who had had an induced abortion (211) or a prior delivery (801). The adolescents with prior deliveries presented with greater perinatal (OR=2.35) and neonatal (OR=4.70) risks and mortality, when compared to the nulliparous participants. The adolescents with a prior abortion presented with higher risks of stillborns (OR=3.31), premature deliveries (OR=2.21), and a very low birth weight (VLBW) (OR=2.74) than the nulliparous adolescents.^(24)^

Researchers established the temporal changes in maternal age and their impacts on the annual rate of cesarean sections and LBW by means of 91,699 data contained in an information system of Obstetrics of the National Hospital of Muhimbili, located in Dar es Salaam (Tanzania), during the period from 1999 to 2005. Based on the results, it was possible to observe that the proportion of adolescent mothers (12 to 19 years) diminished progressively over time, while that of 30 to 34 years increased. As of 1999, the risk of LBW declined, and the risk of cesarean sections increased continually up to a maximum in 2005, but the risk in adolescent mothers was lower than in mothers aged between 35 and 50 years.^(26)^

One study investigated the relation between the first and the second pregnancy in adolescence with premature births, birth weight, and SGA, compared to adult mothers, and showed that the adolescents had a greater risk of premature birth and reduced weight of the newborn when compared to the adult mothers, especially during a second gestation.^(28)^

A study conducted by means of the application of questionnaires and medical record analysis assessed the association between teen pregnancy and LBW in 537 adolescent mothers (10 to 19 years) and in 1,441 adult mothers (20-34 years), showing that the outcomes LBW and prematurity (OR=29.0) were associated with a low number of prenatal visits (OR=2.98), late initiation of prenatal care (OR=1.91), and low level of schooling (OR=1.95). There was a lower incidence of cesarean sections in adolescents (33.3%) than in adults (49.4%), and a lower association with pre-eclampsia and cephalopelvic disproportion.^(25)^

Another study also showed an association between adolescent pregnancy and the late start of prenatal care (OR=1.86) and lower number of visits (OR=2.03).^(27)^

Among the adolescents, also verified was a greater risk of prematurity (OR=1.46) and LBW (OR=1.47), besides the use of an abortive agent at the beginning of gestation (OR=2.34), and among women of an advanced age, a strong association was found between pregnancy and *diabetes mellitus* (OR=9.00), pre-eclampsia (OR=4.38), premature rupture of the membranes (OR=5.81), and higher frequency of cesarean sections (60.3%).^(27)^

One study demonstrated that the chances of LBW (OR=2.70) and of prematurity (OR=5.82) were reduced when the adolescent had six or more prenatal visits.^(34)^ Another study revealed that appropriate prenatal care decreased the chances of fetal death.^(30)^

### Prevalence of gestation in adolescence

Regarding participant inclusion criteria, four studies selected only adolescents, while the others^(11)^ included adolescent and adult and/or advanced age mothers. Of these, eight were studies with a cross-sectional design and were used to calculate the prevalence of gestation in adolescence (Table 1). The national studies demonstrated a prevalence of 26.4% (1,623/6,149).

**Table 1:** Prevalence of pregnancy in adolescence as per the studies included

Reference	Adolescent/population	Incidence (%)
Nili et al.^(20)^	99/2,357	4.2
Santos et al.^(25)^	537/1,978	27.2
Muganyizi et al.^(26)^	16,573/91,699	18.1
Santos et al.^(27)^	549/2,196	25.0
Mukhopadhyay et al.^(29)^	350/700	50.0
Oliveira et al.^(30)^	1,989/9,041	22.0
Lopoo^(32)^	125,796/1,355,962	9.3
Martins et al.^(33)^	537/1,975	27.2

Total	146,430/1,465,908	10.0

### Maternal and neonatal complications related to adolescent gestation

The most often described maternal complications in the selected studies were abortion, pregnancy-induced hypertension, hemorrhagic syndromes, urinary infection, and premature rupture, which are described on table 2. The prevalence of cesarean sections in this population was 26.7% (530/1,983).^(20,23,25,33,34)^

**Table 2:** Maternal complications in adolescent pregnancy, as per the studies included

References	Abortion	HDP	Hemorrhagic syndromes	UTI	PROM
	n (%)	n (%)	n (%)	n (%)	n (%)
Nili et al.^(20)^	-	7/99 (7.1)	-	2/99 (2.0)	20/99 (20.2)
Kongnyuy et al.^(23)^	-	41/268 (15.3)			12/268 (4.5)
Santos et al. ^(25)^	43/537 (8.0)	47/537 (8.8)	4/537 (0.74)	-	-
Santos et al.^(27)^	191/549 (34.8)	50/549 (9.1)	4/549 (0.73)	94/549 (17.1)	6/549 (1.1)
Correia et al.^(31)^	149/2.592 (5.8)	-	-	-	-

Total	383/3.678 (10.4)	145/1.453 (10.0)	8/1.086 (0.74)	96/648 (14.8)	38/916 (4.2)

HDP: hypertensive disorders of pregnancy (pre-eclampsia, eclampsia, and HELLP); UTI: urinary tract infection; PROM: premature rupture of fetal membranes.

Most of the studies focused on verifying the relation between complications in pregnancy and prematurity and LBW among adolescent mothers, correlating them with perinatal and/or neonatal death (Table 3).

**Table 3:** Prevalence of preterm delivery, low birth weight, and perinatal death in adolescents according to the studies included

Reference	PT	LBW	Neonatal death	Perinatal death
	(%)	(%)	(%)	(%)
Nili et al.^(20)^	39/99 (39.4)	33/99 (33.3)	7/99 (7.1)	12/99 (12.1)
Markovitz et al.^(21)^	2.751/29.085 (9.5)	2.613/29.085 (9.0)	161/29.085 (0.6)	-
Kongnyuy et al.^(23)^	57/268 (21.3)	46/268 (17.2)	13/268 (4.9)	17/268 (6.34)
Reime et al.^(24)^	324/8.857 (3.7)	124/8.857 (1.4)	24/8.857 (0.3)	73/8.857 (0.82)
Santos et al.^(25)^	115/537 (21.4)	107/537 (19.9)	-	-
Santos et al.^(27)^	120/549 (21.9)	113/549 (20.6)	-	-
Khashan et al.^(28)^	749/11.142 (6.7)	679/11.142 (6.1)	-	-
Mukhopadhyay et al.^(29)^	97/350 (27.7)	137/350 (39.1)	18/350 (5.1)	36/350 (10.3)
Martins et al.^(33)^	115/537 (21.4)	107/537 (19.9)	-	-
Santos et al.^(34)^	82/542 (15.1)	62/542 (11.4)	-	-

Total	4.449/51.966 (8.6)	4.021/51.966 (7.7)	223/38.659 (0.6)	138/9574 (1.44)

PT: preterm; LBW: low birth weight.

Two studies^(20,27)^ that evaluated the need for admission to a neonatal intensive care unit (NICU) demonstrated that 18.4% (119/648) of the newborns of adolescent mothers were transferred to the NICU. Only one study described the prevalence of infant death in this population,^(21)^ which was approximately 9.6 per thousand liveborns.

## DISCUSSION

Over the last decades, much has been discussed about adolescence, with a greater emphasis on its complexity and its repercussions on pregnancy during this phase. Pregnancy in adolescence is considered a public health problem that should be considered in a comprehensive manner, in order to involve the adolescent mother and the problems that surround her.^(35)^

Nevertheless, the consideration of pregnancy during this stage as a risk factor for adverse outcomes is an oversimplification, since the phenomenon occurs in a variety of transactions and vulnerability, both of the mother and child, may be diminished by means of protective factors.^(36)^ In this way, it becomes evident that not every pregnancy in adolescence carries a high obstetric risk.^(37)^

Among the risk factors reported in pregnancy, low level of schooling, age under 15 years at the first sexual intercourse, absence of a partner, the maternal history of pregnancy in adolescence, and the lack of knowledge and access to contraceptive methods stood out as most significant.^(38)^ Added to these, there is school drop-out, absence of future plans, low self-esteem, alcohol and drug abuse, lack of knowledge as to sexuality, and inappropriate use of contraceptive methods.^(39)^

These factors may influence the adverse reproductive events in reference to the adolescent mother, and should be taken into consideration by the public health programs during preparation of strategies for preventing pregnancy in adolescence.^(38)^

We point out that gestation during adolescence generates serious consequences for the two aspects of mother and child, such as, lack of care and abandonment of the child; emotional problems; school drop-out; job loss or a decline in options of growing in the work market; and multiparity within a short period of time.^(40)^

One study^(26)^ showed that the proportion of adolescent mothers decreased progressively during the period of 1999 to 2005. In Brazil, data from the Ministry of Health describe a 20% prevalence of adolescent gestations. In 2011, of the 2,913,160 liveborns, 560,889 (19.2%) were from adolescent mothers, in which 27,786 were under the age of 15 years.^(41)^

The national studies shown here^(20,25-27,29-30,32-33)^ demonstrated a prevalence significantly greater than that presented by the Ministry of Health, probably due to such studies having been conducted in tertiary services with a greater prevalence of high-risk pregnancies: 26.4% (1,623/6,149) *versus* 19.2% (560,889/2,913,160), with p<0.0001.

In literature in general, some authors demonstrated an increase in maternal-fetal complications at all stages of the gestational cycle among adolescent mothers.^(7,8,42,43)^ In the present study, we observed that the complications associated with adolescent pregnancy most recurrent in literature were more often associated with the newborn than with the mother herself, with a predominance of articles emphasizing prematurity, LBW, and mortality. The occurrence of premature births, low-weight newborns, or infants with very low weight and mortality was significantly greater among babies of adolescent mothers.^(20,21,23-25,27-29,33,34)^

These complications may be correlated with the low number of prenatal visits, late initiation of prenatal care, inappropriate prenatal care, and other factors, such as race, marital status, low level of schooling, smoking, and poverty. Santos et al.^(34)^ observed a relation of the LBW with pregestational weight, pregestational body mass index, and gestational weight gain.

Supplementary literature suggests that the socioeconomic and cultural environments in which the young mother is inserted are associated with the increased frequency of low-weight and premature newborns. Additionally, it is known that prenatal care tends to be inadequate among adolescent mothers,^(44)^ which shows the importance of prenatal visits to decrease complications of pregnancy in this age group.

Adolescent pregnancy is one of the three reproductive variables associated with greater infant mortality, primarily because it is related to a complex interaction of determining factors. In the study by Oliveira et al.,^(30)^ the presence of maternal comorbidity increased the risk of fetal and postnatal deaths. One should point out that most deaths could be avoidable and that the main failures are found in the quality of prenatal care, delivery, and neonatal care.^(45)^

Analyzing the maternal complications related to pregnancy, the present systematic review found a smaller quantity of papers related to the topic^(20,23,25,31)^ when compared to the data from fetal complications. The following complications were cited: pre-eclampsia, eclampsia, HELLP, abortion, urinary infection, and premature rupture of the ovarian membranes, among others. In general, the papers evaluated confirmed that an adolescent would be more inclined to an increase in maternal complications than would an adult pregnant woman.

The presence of comorbidity during the gestational period such as, for example, hypertension, urinary tract infection, pathological vaginal discharge, is much more common among adolescents than at other ages.^(46)^

On the other hand, the prevalence of cesarean sections in this group was significantly lower when compared to the adult population. Data from the Information Technology Department of the Unified Healthcare System (DATASUS, *Dados do Departamentode Informática dos Sistema Único de Saúde*) revealed that between 2008 and 2011, the prevalence of cesarean sections was 50.7% in the general population, and 43% in the adult population.^(41)^

As to the presence of pre-eclampsia and eclampsia as complications of adolescent pregnancy, the results were inconclusive due to divergent data of the selected articles. Some studies declared that it was significantly greater among infants of adolescent mothers.^(20,23)^ However, others observed a smaller association in adolescent mothers.^(25,27)^

Of the articles analyzed, three papers cited abortion as a risk in early pregnancy, emphasizing the expression of not desiring the child, not taking the pregnancy to full-term.^(25,27,31)^ Santos et al.^(27)^ verified a 2.34 risk of the use of an abortive agent at the beginning of gestation in pregnant adolescents. Correia et al.^(31)^ verified that 26.7% of the adolescents had abortions, primarily for fear of their families.

When analyzing specifically the occurrence of abortion in the population of puerperal women, Abreu^(47)^ found a 54.7% proportion of adolescents with antecedents of abortion and identified that most were aged over 16 years (89.7%). As to the quantity of abortions, the same author observed the occurrence of two episodes of abortion, at most, among the adolescents.

It is known that adolescents who get an abortion suffer from lack of information, deficient medical care, of loneliness, and of a lack of communication in the family.^(48)^ Additionally, abortion is responsible for increased hospital admissions, and can result in physical and psychological complications for the mother, and even death.^(49,50)^ According to Granja et al., about 22% of maternal deaths in reference to pregnant adolescents had as primary causes pregnancy-induced hypertension, puerperal sepsis, and septic abortion, representing 75% of the total number of deaths.^(51)^

Urinary infection was cited by two studies of the present systematic review.^(20,27)^ A study by Nili et al.^(20)^ showed that only 2% of the pregnant teens presented with urinary tract infections. In the study by Santos et al.,^(27)^ in urinary infection occurred in 17.1% of the adolescents. Just as the present systematic review, the study had the objective of establishing the profile of pregnancy in adolescence in a population cared for by the Unified Healthcare System (SUS, *Sistema Único de Saúde*) in the city of Muriaé, in the region *Zona da Mata Mineira*, and verified that urinary infection was one of the most frequent complications among the adolescent puerpera, occurring with a greater proportion in adolescents over 16 years of age.^(47)^

As to complications of the delivery, the occurrence of premature rupture of the membranes was described in three articles.^(20,23,27)^ One study reported a 20.3% frequency of premature rupture in pregnant adolescents, suggesting that nutritional deficiencies may play an important role in this complication.^(20)^

Yazlle et al. reported obstetric complications in 38.3% of the adolescents and among the most frequent diagnoses were problems with the fetus or the placenta, and problems with the amniotic cavity and membranes.^(52)^

Despite the increase in coverage for this population given by Primary Care, we noted gaps in the health education and prevention programs that stimulate the use of male and female contraceptive agents, besides the inexistence of public policies directed at young pregnant women.^(38)^ These factors collaborate towards the lack of knowledge of the adolescent about prevention methods and the appearance of an undesired pregnancy and its possible complications. Within this context, the need for prevention and control of consequences of an early pregnancy is justified.^(40)^

Therefore, it is up to the healthcare professionals to improve listening, strengthen bonds with the adolescents, guarantee access to information and to contraceptive measures, and promote collective actions that help adolescents deal with their sexuality and develop self-care, and to also increase access to educational and recreational activities.^(53)^

It is important to point out that this study has some limitations. Only the MEDLINE (PubMed), LILACS, and SciELO databases were used, *i.e*., those considered most important in the field of health. However, other databases could have been consulted, such as EMBASE: Biomedical Answers, EBSCO, and SCOPUS. Additionally, in selected cross-sectional studies is it difficult to establish with precision the cause-effect relation, since the causal relation may suffer influences from confounding factors. The scarcity of data from good quality randomized controlled clinical studies to evaluate the complications of pregnancy in adolescence was also a limiting factor.

## CONCLUSIONS

The main neonatal complications found were prematurity, low or very low birth weight, and perinatal mortality. Whereas the major maternal complications were hypertensive pregnancy disorders, abortion, urinary infections, and premature rupture of the fetal membranes. However, it is important to point out that the data are controversial as to the occurrence of pre-eclampsia.

Within this context, the importance of conducting studies for further clarification as to neonatal mortality, which seems to be strongly influenced by some determinants, such as low birth weight and prematurity, as well as maternal complications related to adolescent pregnancy. This fact reinforces the importance of prevention of these variables in prenatal and delivery care.

## References

[1] Futterman D, Chabon B, Hoffman ND (2000). HIV and AIDS in adolescents. Pediatr Clin North Am.

[2] Instituto Brasileiro de Geografia e Estatística (IBGE), Diretoria de Pesquisas, Coordenação da População e Indicadores Sociais (2009). Indicadores sociodemográficos e de saúde no Brasil.

[3] Brasil, Ministério da Saúde, DATASUS, DATASUS (2012). Informações de saúde. Sistema de informações sobre nascidos vivos.

[4] Azevedo DV, Sampaio HA (2003). Fatores de risco associados à gestação na adolescência. Femina.

[5] Elfenbein DS, Felice ME (2003). Adolescent pregnancy. Pediatr Clin North Am.

[6] Carvalho RC, Campos H de H, Bruno ZV, Mota RM (2006). Fatores preditivos de hipertensão gestacional em adolescentes primíparas: análise do pré-natal, da MAPA e da microalbuminúria. Arq Bras Cardiol.

[7] Michelazzo D, Yazlle ME, Mendes MC, Patta MC, Rocha JS, Moura MD (2004). Indicadores sociais de grávidas adolescentes: estudo caso-controle. Rev Bras Ginecol Obstet.

[8] Iacobelli S, Robillard PY, Gouyon JB, Hulsey TC, Barau G, Bonsante F (2012). Obstetric and neonatal outcomes of adolescent primiparous singleton pregnancies: a cohort study in the South of Reunion Island, Indian Ocean. J Matern Fetal Neonatal Med.

[9] Aquino-Cunha M, Queiroz-Andrade M, Tavares J, Andrade T (2002). Gestação na adolescência: relação com o baixo peso ao nascer. Rev Bras Ginecol Obstet.

[10] Rocha RC, Souza E, Guazzelli CA, Chambô A, Soares EP, Nogueira ES (2006). Prematuridade e baixo peso entre recém-nascidos de adolescentes primíparas. Rev Bras Ginecol Obstet.

[11] Chalem E, Mitsuhiro SS, Ferri CP, Barros MC, Guinsburg R, Laranjeira R (2007). Gravidez na adolescência: perfil sócio-demográfico e comportamental de uma população da periferia de São Paulo, Brasil. Cad Saude Publica.

[12] Clarke M, Horton R (2001). Bringing it all together: Lancet-Cochrane collaborate on systematic reviews. Lancet.

[13] Clarke M, Rev Man (2004). Formulating the problem. Cochrane review’s handbook.

[14] Taminato M, Fram D, Torloni MR, Belasco AG, Saconato H, Barbosa DA (2011). Screening for group B Streptococcus in pregnant women: a systematic review and meta-analysis. Rev Lat Am Enferm.

[15] Moher D, Jadad AR, Klassen TP (1998). Guides for reading and interpreting systematic reviews: III. How did the authors synthesize the data and make their conclusions?. Arch Pediatr Adolesc Med.

[16] Stocco JG (2009). Avaliação de complicações infecciosas relacionadas ao uso de catéter venoso central em recém-nascidos e crianças: revisão sistemática.

[17] von Elm E, Altman DG, Egger M, Pocock SJ, Gøtzsche PC, Vandenbroucke JP, STROBE Initiative (2008). The Strengthening the Reporting of Observational Studies in Epidemiology (STROBE) statement: guidelines for reporting observational studies. J Clin Epidemiol.

[18] Malta M, Cardoso LO, Bastos FI, Magnanini MM, Silva CM (2010). STROBE initiative: guidelines on reporting observational studies. Rev Saude Publica.

[19] Mendes KG, Theodoro H, Rodrigues AD, Olinto MT (2012). Prevalência da síndrome metabólica e seus componentes na transição menopáusica: uma revisão sistemática. Cad Saude Publica.

[20] Nili F, Rahmati MR, Sharifi SM (2002). Maternal and neonatal outcome in teenage pregnancy in Tehran Valiasr Hospital. Acta Med Iran.

[21] Markovitz BP, Cook R, Flick LH, Leet TL (2005). Socioeconomic factors and adolescent pregnancy outcomes: distinctions between neonatal and post-neonatal deaths?. BMC Public Health.

[22] Stewart CP, Katz J, Khatry SK, LeClerq SC, Shrestha SR, West KP (2007). Preterm delivery but not intrauterine growth retardation is associated with young maternal age among primiparae in rural Nepal. Matern Child Nutr.

[23] Kongnyuy EJ, Nana PN, Fomulu N, Wiysonge SC, Kouam L, Doh AS (2008). Adverse perinatal outcomes of adolescent pregnancies in Cameroon. Matern Child Health J.

[24] Reime B, Schücking BA, Wenzlaff P (2008). Reproductive outcomes in adolescents who had a previous birth or an induced abortion compared to adolescents’ first pregnancies. BMC Pregnancy Childbirth.

[25] Santos GH, Martins M da G, Sousa M da S (2008). Gravidez na adolescência e fatores associados com baixo peso ao nascer. Rev Bras Ginecol Obstet.

[26] Muganyizi PS, Kidanto HL (2009). Impact of change in maternal age composition on the incidence of Caesarean section and low birth weight: analysis of delivery records at a tertiary hospital in Tanzania,1999-2005. BMC Pregnancy Childbirth.

[27] Santos GH, Martins M da G, Sousa M da S, Batalha S de J (2009). Impacto da idade materna sobre os resultados perinatais e via de parto. Rev Bras Ginecol Obstet.

[28] Khashan AS, Baker PN, Kenny LC (2010). Preterm birth and reduced birthweight in first and second teenage pregnancies: a register-based cohort study. BMC Pregnancy Childbirth.

[29] Mukhopadhyay P, Chaudhuri RN, Paul B (2010). Hospital-based perinatal outcomes and complications in teenage pregnancy in India. J Health Popul Nutr.

[30] Oliveira EF, Gama SG, Silva CM (2010). Gravidez na adolescência e outros fatores de risco para mortalidade fetal e infantil no município do Rio de Janeiro, Brasil. Cad Saude Publica.

[31] Correia DS, Cavalcante JC, Egito ES, Maia EM (2011). Prática do abortamento entre adolescentes: um estudo em dez escolas de Maceió (AL, Brasil). Cien Saude Colet.

[32] Lopoo LM (2011). Labor and delivery complications among teenage mothers. Biodemography Soc Biol.

[33] Martins M da G, Santos GH, Sousa M da S, Costa JE, Simões VM (2011). Associação de gravidez na adolescência e prematuridade. Rev Bras Ginecol Obstet.

[34] Santos MM, Baião MR, Barros DC, Pinto AA, Pedrosa PM, Saunders C (2012). Estado nutricional pré-gestacional, ganho de peso materno, condições da assistência pré-natal e desfechos perinatais adversos entre puérperas adolescentes. Rev Bras Epidemiol.

[35] Moreira TM, Viana D de S, Queiroz MV, Jorge MS (2008). Conflitos vivenciados pelas adolescentes com a descoberta da gravidez. Rev Esc Enferm USP.

[36] Cerqueira-Santos E, Paludo SS, dei Schiro ED, Koller SH (2010). Gravidez na adolescência: análise contextual de risco e proteção. Psicol Estud.

[37] Bouzas I, Miranda AT (2004). Gravidez na adolescência. Adolesc Saude.

[38] Amorim MM, Lima L de A, Lopes CV, Araújo DK, Silva JG, César LC (2009). Fatores de risco para a gravidez na adolescência em uma maternidade-escola da Paraíba: estudo caso-controle. Rev Bras Ginecol Obstet.

[39] Rodrigues RM (2010). Gravidez na adolescência. Nascer e Crescer.

[40] Lima LS, Tocci HA (2001). Gravidez na adolescência: intercorrências e prematuridade. Rev Enferm UNISA.

[41] Brasil, Ministério da Saúde, DATASUS, Informações de Saúde (TABNET) (2011). Estatística Vitais.

[42] Del Ciampo LA, Junqueira MJ, Ricco RG, Daneluzzi JC, Ferraz IS, Martinelli CE (2004). Tendência secular da gravidez na adolescência. Pediatr (São Paulo)..

[43] Fiorelli LR, Krebs VL (2006). Características clínicas e morbidade de recém-nascidos filhos de mães adolescentes em hospital universitário. Rev Med (São Paulo)..

[44] Costa EL, Sena MC, Dias A (2011). Gravidez na adolescência: determinante para prematuridade e baixo peso. Com Ciencias Saude.

[45] Lansky S, França E, Leal MC (2002). Mortalidade perinatal e evitabilidade: revisão da literatura. Rev Saude Publica.

[46] Victora CG (2001). Intervenções para reduzir a mortalidade infantil pré-escolar e materna no Brasil. Rev Bras Epidemiol.

[47] Abreu CW (2010). Aspectos obstétricos, sócio-demográficos e psicossociais de puérperas adolescentes assistidas pelo sistema de saúde do município de Muriaé - Zona da Mata Mineira, Brasil.

[48] Costa TJ (2002). Gravidez na adolescência: um estudo de caso sobre a maternidade na faixa etária de 10 a 14 anos em Juiz de Fora, MG.

[49] Quiala MB, Ojeda SO, Durruti RD, Infante MM (1999). Respuesta del estado psicológico en adolescentes con interrupción del embarazo. Rev Cuba Enferm.

[50] Vieira LM, Goldberg TB, Saes S de O, Dória AA (2007). Abortamento na adolescência: um estudo epidemiológico. Cien Saude Colet.

[51] Granja AC, Machungo F, Gomes A, Bergström S (2001). Adolescent maternal mortality in Mozambique. J Adolesc Health.

[52] Yazlle ME, Franco RC, Michelazzo D (2009). Gravidez na adolescência: uma proposta para prevenção. Rev Bras Ginecol Obstet.

[53] Gurgel MG, Alves MD, Vieira NF, Pinheiro PN, Barroso GT (2008). Gravidez na adolescência: tendência na produção científica de enfermagem. Esc Anna Nery Rev Enferm.

