# Supplementary material for: Complications in adolescent pregnancy: systematic review of the literature
Source: Einstein (Sao Paulo). 2015 Oct-Dec;13(4):618–26. doi: 10.1590/S1679-45082015RW3127 (PMC4878642; doi:10.1590/S1679-45082015RW3127)
Supplement: Translation

# Translation: Complicações da gravidez na adolescência: revisão sistemática da literatura

**Authors:** Walter Fernandes de Azevedo, Michele Baffi Diniz, Eduardo Sérgio Valério Borges da Fonseca, Lícia Maria Ricarte de Azevedo, Carla Braz Evangelista

**Affiliations:** 1 Universidade Federal da Paraíba, João Pessoa, PB, Brasil.; 2 Universidade Cruzeiro do Sul, São Paulo, SP, Brasil.

**Keywords:** Adolescente, Gravidez, Gravidez na adolescência, Complicações na gravidez

## Abstract

A atividade sexual na adolescência pode levar a uma gravidez indesejável que por sua vez pode trazer sérias complicações maternais e fetais. O presente estudo teve por objetivo avaliar as complicações relacionadas à gravidez na adolescência, por meio de uma revisão sistemática, utilizando como descritores do *Medical Subject Headings:* “*pregnancy complication*” *AND* “*adolescent*” *OR* “*pregnancy in adolescence*”. Foram considerados os artigos originais completos em inglês ou português, na íntegra, que apresentassem descrição clara da metodologia. Não foram incluídos estudos qualitativos, revisões ou metanálises, editoriais, série de casos e relatos de caso. A amostra foi constituída por 15 publicações, sendo 10 com delineamento transversal e 5 com delineamento coorte. A prevalência geral de gestação na adolescência foi de 10% e, entre os trabalhos nacionais, de 26%. A prevalência de parto cesárea foi menor que a descrita na população geral. As principais complicações maternas e neonatais de mães adolescentes foram doença hipertensiva específica da gestação, prematuridade e baixo peso ao nascer respectivamente. A gestação na adolescência se relacionou a maior frequência de complicações neonatais e maternas e à menor prevalência de parto cesariana.

## INTRODUÇÃO

A atividade sexual, na adolescência, inicia-se cada vez mais precocemente, com consequências indesejáveis imediatas, como o aumento da frequência de doenças sexualmente transmissíveis (DST) e gravidez, muitas vezes também indesejável, e que, por isso, pode terminar em abortamento.^(1)^

No Brasil, no período de 2000 a 2006, o Sistema de Informação sobre Nascidos Vivos (SINASC) registrou declínio da participação dos nascimentos oriundos de mães dos grupos etários de 15 a 19 anos. Entretanto, a proporção de nascidos vivos cujas mães pertenciam ao grupo etário inferior a 14 anos se manteve estável. Em 2006, 51,4% dos nascidos vivos eram filhos de mães com idade até 24 anos, sendo aproximadamente 1% de mães do grupo etário inferior a 14 anos; 20,6% de mães com idade de 15 a 19 anos; e 29,9% de mães com idade entre 20 e 24 anos.^(2)^ Em 2012, dos 2.905.789 nascidos vivos, 560.147 (19,28%) foram de mães adolescentes.^(3)^

Do ponto de vista biológico, dentre as consequências da gravidez para a adolescente, citam-se maiores incidências de síndrome hipertensiva da gravidez (SHG), anemia, diabetes gestacional, complicações no parto, determinando aumento da mortalidade materna e infantil.^(4-6)^ É importante notar que alguns estudos têm demonstrado aumento na incidência de intercorrências pré-natais, intraparto e pós-parto entre gestantes adolescentes.^(7,8)^

No tocante aos problemas com o recém-nascido, a gravidez na adolescência está associada a taxas mais elevadas de baixo peso ao nascer (BPN), parto pré-termo, doenças respiratórias e tocotraumatismo, além de maior frequência de complicações neonatais e mortalidade infantil.^(9-11)^

Considerando a alta prevalência da gestação na adolescência e suas consequências, o estudo teve o objetivo de avaliar as complicações relacionadas à gravidez na adolescência.

## MÉTODOS

Trata-se de um estudo de revisão sistemática da literatura, que seguiu as recomendações propostas pela Colaboração Cochrane.^(12,13)^

A questão norteadora proposta para o estudo foi a seguinte: Quais as complicações relacionadas à gravidez na adolescência?

A coleta de dados ocorreu entre os meses de maio e agosto de 2012 mediante busca *on-line *nas seguintes bases de dados consultadas a partir da Biblioteca Virtual de Saúde (BVS): MEDLINE (PubMed), Literatura Latino-Americana e do Caribe de Informação em Ciências da Saúde (LILACS) e *Scientific Electronic Library Online* (SciELO).

Para a localização dos artigos, foram utilizados os seguintes descritores do *Medical Subject Headings* (MeSH), da base PubMed/MEDLINE: “*pregnancy complication*” *AND “adolescent” OR “pregnancy in adolescence”*.

Os critérios de inclusão contemplaram artigos originais, disponíveis na íntegra gratuitamente na versão *on-line*, nos idiomas inglês ou português, e no período de 2002 a 2012. Foram incluídos ensaios clínicos randomizados, ensaios clínicos quase randomizados, ensaios clínicos aleatórios, estudos observacionais analíticos (estudos de caso-controle, coorte prospectivos e retrospectivos) e estudos descritivos transversais (de prevalência), que apresentavam uma descrição clara da metodologia. Estudos realizados com grandes amostras de adultos, mas que incluíam adolescentes como um subgrupo, também foram incluídos.

Não foram incluídos artigos teóricos, investigações com uma descrição pouco clara sobre a metodologia utilizada, manuscritos baseados em relatórios estatísticos anuais (dados censitários e informações obtidas de forma indireta por meio de gráficos ou arquivos), estudos qualitativos, revisões ou metanálises, teses e dissertações, editoriais, artigos de opinião, série de casos, relatos de caso, estudos com amostra não representativa de uma população e anteriores ao ano 2002.

Os estudos foram inicialmente estratificados de acordo com os tipos de desenhos e, posteriormente, em relação aos desfechos, seguindo a metodologia Cochrane.^(14)^

A qualidade metodológica da revisão sistemática foi definida com a confiança de que o desenho e o relato do estudo estivessem livres de bias,^(15)^ e foi avaliada independentemente por dois revisores, no sentido de se averiguar se preencheram os critérios de inclusão e exclusão. Em caso de dúvida ou discordância, foi solicitado a um terceiro revisor a emissão de parecer sobre o estudo ser ou não incluído, conforme propõe Stocco.^(16)^ No caso de estudos duplicados, foi incluído o mais recente ou com informações mais completas.

Para avaliação da qualidade metodológica, critérios de inclusão e exclusão, foram utilizados as recomendações STROBE (*The Strengthening the Reporting of Observational Studies in Epidemiology*).^(17,18)^ A avaliação foi dividida em três categorias de estudos: (A) nos casos de estudos que preenchiam valor ≥80% dos critérios solicitados; (B) nos casos que preenchiam de 79 a 50% dos critérios; e (C) nos casos que preenchiam <50% dos critérios estabelecidos.^(14,17,18)^ Assim, apenas os artigos que atingiram um percentual >50% (classificados como A ou B) foram considerados de boa qualidade e incluídos na pesquisa.^(19)^

Os dados analisados foram sintetizados e organizados por meio de figuras, quadros e tabelas.

## RESULTADOS 

O universo foi constituído por 6.465 artigos, sendo 6.232 no PubMed/MEDLINE e 233 na LILACS e SciELO. Após a leitura dos títulos e/ou resumos, foram excluídos 6.380 artigos, por apresentarem foco diferente do objetivo procurado. Assim, das 85 publicações lidas na íntegra, foram selecionadas 15, que se enquadraram nos critérios de inclusão e exclusão, conforme a figura 1.

**Figura 1:** Fluxograma de identificação e seleção dos estudos da revisão sistemática

O quadro 1 mostra os países de origem, ano de publicação e desenho dos estudos incluídos na presente revisão.

**Quadro 1:** Relação dos estudos incluídos de acordo com os países de origem e o delineamento do estudo

Referência	País	Delineamento do estudo	STROBE
Nili et al.^(20)^	Irã	Transversal	B
Markovitz et al.^(21)^	Estados Unidos	Coorte retrospectivo	B
Stewart et al.^(22)^	Nepal	Coorte retrospectivo	A
Kongnyuy et al.^(23)^	Camarões	Coorte prospectivo	B
Reime et al.^(24)^	Alemanha	Coorte retrospectivo	A
Santos et al.^(25)^	Brasil	Transversal	B
Muganyizi et al.^(26)^	Tanzânia	Transversal	B
Santos et al.^(27)^	Brasil	Transversal	B
Khashan et al.^(28)^	Inglaterra	Coorte retrospectivo	A
Mukhopadhyay et al.^(29)^	Índia	Transversal	B
Oliveira et al.^(30)^	Brasil	Transversal	B
Correia et al.^(31)^	Brasil	Transversal	B
Lopoo^(32)^	EUA	Transversal	B
Martins et al.^(33)^	Brasil	Transversal	B
Santos et al.^(34)^	Brasil	Transversal	B

Dos 15 trabalhos selecionados, dez apresentaram delineamento transversal e 5 apresentaram coorte, sendo 4 retrospectivos e 1 prospectivos. Quanto ao ano de publicação, a maioria dos estudos foi publicada no período de 2008 a 2012. Os estudos foram realizados predominantemente no Brasil, Estados Unidos, nos países da Europa, África e Ásia. Todos os artigos selecionados para a revisão sistemática apresentaram percentuais STROBE >50%, sendo 3 classificados como STROBE A e 12 como STROBE B.

O quadro 2 apresenta as principais características dos estudos acerca das complicações da gravidez na adolescência.

**Quadro 2:** Características dos estudos incluídos sobre as complicações da gravidez na adolescência (2002/2012)

Referência	Objetivo	Principais desfechos
Nili et al.^(20)^	Determinar a frequência de adolescentes grávidas e as complicações maternas e neonatais.	Complicações maternas: pré-eclâmpsia, RPM, ITU e doença cardíaca e na tireoide. Complicações neonatais: prematuridade, BPN, crescimento intrauterino retardado e mortalidade.
Markovitz et al.^(21)^	Investigar a relação entre a mortalidade infantil, nível socioeconômico e idade materna.	Riscos de mortalidade infantil, neonatal e pós-neonatal foram significativamente maiores para adolescentes mais jovens.
Stewart et al.^(22)^	Determinar a associação entre idade materna jovem e desfechos adversos.	A idade materna jovem foi associada com o aumento do risco de prematuridade entre as primíparas.
Kongnyuy et al.^(23)^	Determinar os desfechos fetais adversos associados à gravidez na adolescência.	Os desfechos fetais foram BPN, prematuridade e óbito neonatal. Os desfechos maternos foram eclâmpsia, pré-eclâmpsia, laceração perineal e episiotomia.
Reime et al.^(24)^	Comparar o risco de desfechos adversos em adolescentes nulíparas e que tiveram aborto induzido ou parto anterior.	As adolescentes com parto anterior apresentaram maiores riscos perinatais, neonatais e mortalidade. As com aborto anterior apresentaram riscos elevados para natimortos, prematuridade e MBPN.
Santos et al.^(25)^	Analisar a associação da gravidez na adolescência com o BPN.	Entre as adolescentes, o BPN e a prematuridade estiveram associados com a baixa escolaridade, baixo número de consultas do pré-natal e início tardio do pré-natal.
Muganyizi et al.^(26)^	Estabelecer as mudanças temporais na idade materna e seus impactos para cesarianas e BPN.	A proporção de mães adolescentes diminuiu progressivamente ao longo do tempo (1999-2005). O risco de cesariana aumentou e o risco de BPN foi reduzido.
Santos et al.^(27)^	Analisar a associação entre idade materna, resultados perinatais e via de parto.	Entre as adolescentes, foi verificado maior risco de prematuridade, BPN, frequência de usos de abortivos, baixo número de consultas do pré-natal e início tardio.
Khashan et al.^(28)^	Investigar a relação entre a primeira e segunda gravidez na adolescência e parto pré-termo, peso ao nascer e PIG.	O risco de nascimento prematuro foi aumentado na primeira e segunda gestação. Houve alguma evidência de um efeito protetor contra PIG na primeira gestação de adolescentes.
Mukhopadhyay et al.^(29)^	Comparar as diferenças perinatais entre adolescentes e adultas primigestas.	As mães adolescentes apresentaram maior proporção de partos prematuros, BPN e natimortos.
Oliveira et al.^(30)^	Identificar o efeito da gravidez na adolescência e fatores de risco para mortalidade fetal e infantil.	O baixo peso e a prematuridade foram determinantes do óbito fetal e neonatal. O risco de óbito pós-neonatal foi mais elevado na presença de multiparidade, morbidade materna e BPN.
Correia et al.^(31)^	Investigar o aborto em adolescentes.	26,7% das adolescentes abortaram principalmente por medo.
Lopoo^(32)^	Investigar a relação entre gravidez na adolescência e complicações do parto.	As adolescentes tiveram menores taxas de RPMO, placenta prévia, trabalho de parto prolongado, apresentação pélvica, dentre outras complicações.
Martins et al.^(33)^	Analisar a associação da gravidez na adolescência com prematuridade.	Houve uma associação entre a prematuridade e o menor número de consultas no pré-natal, início tardio do pré-natal, BPN e baixa escolaridade.
Santos et al.^(34)^	Identificar a associação do estado nutricional materno e condições do pré-natal com o BPN e prematuridade.	O peso ao nascer foi relacionado ao intervalo intergestacional, ao peso pré-gestacional, ao IMC pré-gestacional e ao ganho de peso gestacional total.

RPM: ruptura prolongada de membranas; ITU: infecção do trato urinário; BPN: baixo peso ao nascer; MBPN: muito baixo peso ao nascer; PIG: pequeno para idade gestacional; RPMO: rotura prematura das membranas ovulares; IMC: índice de massa corporal.

Em pesquisa realizada com 2.357 mulheres grávidas, a frequência de adolescentes grávidas (menores de 18 anos) foi de 4%. A complicação materna que apresentou um maior destaque foi a ruptura prolongada da membrana, com 20,3%, seguida de pré-eclâmpsia (7,1%), doenças na tireoide (7,1%), doenças cardíacas (3%) e infecções no trato urinário (2%). Dentre as complicações neonatais, destacaram-se a prematuridade (39%), o BPN (32%) e o crescimento intrauterino retardado (12%). A mortalidade neonatal foi descrita em 6,9% dos casos, sendo significativamente maior que as complicações neonatais dos demais partos.^(20)^

Outro estudo realizado com 265 mães adolescentes (idade ≤19 anos) e com 832 mães com idade ente 20 e 29 anos mostrou que as complicações maternas relacionadas à gravidez na adolescência mais frequentes foram a eclâmpsia (OR=3,18), a pré-eclâmpsia (OR=1,82), a laceração perineal (OR=1,45) e a episiotomia (OR=1,82). Enquanto que as complicações fetais foram o BPN (OR=1,71), prematuridade (OR=1,77) e óbito neonatal precoce (OR=2,18).^(23)^

Estudo realizado por meio de análise de dados coletados pelo Departamento de Serviço de Saúde do Estado do Texas entre 1994 e 2003 avaliou as complicações que ocorreram durante o trabalho de parto de 1.355.962 mães nulíparas e mostrou que as mães adolescentes (15 a 18 anos) tiveram menores taxas de febre intraparto, mecônio excessivo, ruptura prematura da membrana, placenta prévia, trabalho de parto prolongado, parto disfuncional, apresentação pélvica do feto, desproporção cefalopélvica e prolapso de cordão umbilical, quando comparadas às mães com idade entre 25 e 29 anos.^(32)^

Mukhopadhyay et al. compararam as diferenças perinatais entre 350 adolescentes (13 a 19 anos) e 350 adultas (20 a 29 anos), ambas primigestas, por meio de análise de prontuários, e evidenciaram que houve uma maior proporção de partos prematuros (27,7%), BPN (38,95%) e taxa de natimortos (5,1%), em comparação às mães adultas.^(29)^

Uma pesquisa investigou as razões que levaram adolescentes a provocarem o aborto, relacionando a motivação com idade e tipo de escola que frequentavam. Das 2.592 jovens participantes da pesquisa, 182 (7,0%) referiram ter engravidado e 149 (26,7%) abortado. Verificou-se que o medo da reação dos pais (o mais citado), a idade, a falta de apoio por parte do companheiro e não aceitar a gravidez foram razões que levaram as adolescentes a provocarem o aborto. O aborto obteve uma maior frequência entre as jovens de escola pública.^(31)^

Estudo realizado por meio por meio de análise de dados de residentes no Estado do Missouri (Estados Unidos) durante os anos de 1997 a 1999 investigou a relação entre a mortalidade infantil (neonatal e pós-natal), nível socioeconômico e idade materna. Tal estudo envolveu 10.131 adolescentes entre 12 e 17 anos, 18.954 adolescentes entre 18 e 19 anos e 28.899 adultas (20 a 34 anos) e mostrou que os riscos de mortalidade infantil (OR=1,95), neonatal (OR=1,69) e pós-neonatal (OR=2,47) foram significativamente maiores entre adolescentes de 12 a 17 anos do que entre as adultas (20 a 34 anos). Após o ajuste para raça, estado civil, nível educacional, tabagismo, assistência pré-natal e pobreza, o risco de mortalidade pós-neonatal (OR=1,73) manteve-se significativamente maior para mães adolescentes mais jovens, mas não o risco de mortalidade neonatal (OR=1,43).^(21)^

Pesquisadores determinaram a associação entre idade materna jovem e o risco de BPN, pequeno para a idade gestacional (PIG) e parto prematuro em 1.359 adolescentes nulíparas (759) ou uníparas (600), que tiveram suplementação da dieta com micronutrientes e que pariram uma criança que foi analisada dentro de 72 horas após o parto, no período de 1999 a 2001. Os resultados mostraram que não houve diferença no risco de BPN (OR=0,96) ou PIG (OR=1,01) por ano de aumento da idade materna entre as primíparas. A idade materna jovem não afetou a antropometria e nem a idade gestacional da prole. Entre primíparas, cada ano de aumento da idade materna foi associado ao aumento do comprimento ao nascer, cabeça e circunferência do peito, mas não peso de sua prole. A idade materna jovem (≤18 anos) foi associada com o aumento do risco de parto prematuro entre as primíparas (OR=2,07).^(22)^

Estudo realizado por meio de análise de dados perinatais coletados no período de 1990 a 1999 comparou o risco de desfechos reprodutivos adversos em adolescentes nulíparas (7.845) e adolescentes que tiveram aborto induzido (211) ou parto anterior (801). As adolescentes com parto anterior apresentaram maiores riscos perinatais (OR=2,35), neonatais (OR=4,70) e mortalidade, quando comparadas às nulíparas. As adolescentes com aborto anterior apresentaram riscos mais elevados para natimortos (OR=3,31), nascimentos prematuros (OR=2,21) e muito BPN (OR=2,74) do que as adolescentes nulíparas.^(24)^

Estudiosos procuraram estabelecer as mudanças temporais na idade materna e seus impactos na taxa anual de cesarianas e BPN, por meio de 91.699 dados contidos em um sistema de informação da Obstetrícia do Hospital Nacional de Muhimbili, localizado em Dar es Salaam (Tanzânia), no período de 1999 a 2005. Diante dos resultados, foi possível observar que a proporção de mães adolescentes (12 a 19 anos) diminuiu progressivamente ao longo do tempo, enquanto que a de 30 a 34 anos aumentou. A partir de 1999, o risco de BPN reduziu, e o risco de cesariana aumentou continuamente para um máximo em 2005, porém, o risco em mães adolescentes foi menor que em mães com idades entre 35 e 50 anos.^(26)^

Pesquisa investigou a relação entre a primeira e a segunda gravidez na adolescência com nascimentos prematuros, peso ao nascer e PIG, comparando com mães adultas, e mostrou que as mães adolescentes tinham maior risco de parto prematuro e peso reduzido do bebê quando comparadas com as mães adultas, principalmente em uma segunda gestação.^(28)^

Pesquisa realizada por meio da aplicação de questionários e análise de prontuários analisou a associação da gravidez na adolescência com o BPN em 537 mães adolescentes (10 a 19 anos) e em 1.441 mães adultas (20-34 anos), mostrando que os desfechos BPN e prematuridade (OR=29,0) estiveram associados com baixo número de consultas do pré-natal (OR=2,98), início tardio do pré-natal (OR=1,91) e baixa escolaridade (OR=1,95). Houve menor incidência de cesárea em adolescentes (33,3%) que em adultas (49,4%) e menor associação com pré-eclâmpsia e desproporção cefalopélvica.^(25)^

Outra pesquisa também demonstrou associação entre a gravidez na adolescência e o início tardio do pré-natal (OR=1,86), e o menor número de consultas (OR=2,03).^(27)^

Entre as adolescentes, foi verificado, ainda, maior risco de prematuridade (OR=1,46) e BPN (OR=1,47), além de uso de abortivo no início da gestação (OR=2,34) e, entre mulheres com idade avançada, constatou-se forte associação da gravidez com *diabetes mellitus* (OR=9,00), pré-eclâmpsia (OR=4,38), ruptura prematura de membranas (OR=5,81) e maior frequência de parto cesáreo (60,3%).^(27)^

Estudo demonstrou que as chances de BPN (OR=2,70) e de prematuridade (OR=5,82) reduziram quando a adolescente recebeu seis ou mais consultas de pré-natal.^(34)^ Outro estudo revelou que o pré-natal adequado diminui as chances de óbito fetal.^(30)^

### Prevalência de gestação na adolescência

Em relação aos critérios de inclusão das participantes, quatro estudos selecionaram como participantes apenas adolescentes, sendo que os demais^(11)^ incluíram tanto mães adolescentes como mães adultas e/ou em idade avançada. Destes, oito eram estudos com delineamento transversal e foram utilizados para cálculo da prevalência de gestação na adolescência (Tabela 1). Os estudos nacionais demonstram prevalência de 26,4% (1.623/6.149).

**Tabela 1:** Prevalência de gravidez na adolescência de acordo com os estudos incluídos

Referência	Adolescentes/população	Incidência (%)
Nili et al.^(20)^	99/2.357	4,2
Santos et al.^(25)^	537/1.978	27,2
Muganyizi et al.^(26)^	16.573/91.699	18,1
Santos et al.^(27)^	549/2.196	25,0
Mukhopadhyay et al.^(29)^	350/700	50,0
Oliveira et al.^(30)^	1.989/9.041	22,0
Lopoo^(32)^	125.796/1.355.962	9,3
Martins et al.^(33)^	537/1.975	27,2

Total	146.430/1.465.908	10,0

### Complicações maternas e neonatais relacionadas à gestação na adolescência

As complicações maternas mais descritas nos estudos selecionados foram: abortamento, doença hipertensiva da gestação, síndromes hemorrágicas, infecção urinária e rotura prematura que são descritas na tabela 2. A prevalência de cesárea, nessa população, foi de 26,7% (530/1.983).^(20,23,25,33,34)^

**Tabela 2:** Complicações maternas na gravidez entre adolescentes. De acordo com os estudos incluídos

Referências	Aborto	DHEG	Síndromes hemorrágicas	ITU	RPMO
**n (%)**	**n (%)**	**n (%)**	**n (%)**	**n (%)**
Nili et al.^(20)^	-	7/99 (7,1)	-	2/99 (2,0)	20/99 (20,2)
Kongnyuy et al.^(23)^	-	41/268 (15,3)			12/268 (4,5)
Santos et al.^(25)^	43/537 (8,0)	47/537 (8,8)	4/537 (0,74)	-	-
Santos et al.^(27)^	191/549 (34,8)	50/549 (9,1)	4/549 (0,73)	94/549 (17,1)	6/549 (1,1)
Correia et al.^(31)^	149/2.592 (5,8)	-	-	-	-

Total	383/3.678 (10,4)	145/1.453 (10,0)	8/1.086 (0,74)	96/648 (14,8)	38/916 (4,2)

DHEG: doença hipertensiva específica da gestação (pré-eclâmpsia, eclâmpsia e HELLP); ITU: infecção do trato urinário; RPMO: rotura prematura das membranas ovulares.

A maioria dos estudos preocupou-se em verificar a relação entre as complicações na gravidez com prematuridade e BPN entre mães adolescentes, correlacionando com a morte perinatal e/ou neonatal (Tabela 3).

**Tabela 3:** Prevalência de parto pré-termo, de baixo peso e morte perinatal em adolescentes de acordo com os estudos incluídos

Referência	PT	BPN	Morte neonatal	Morte perinatal
**(%)**	**(%)**	**(%)**	**(%)**
Nili et al.^(20)^	39/99 (39,4)	33/99 (33,3)	7/99 (7,1)	12/99 (12,1)
Markovitz et al.^(21)^	2.751/29.085 (9,5)	2.613/29.085 (9,0)	161/29.085 (0,6)	-
Kongnyuy et al.^(23)^	57/268 (21,3)	46/268 (17,2)	13/268 (4,9)	17/268 (6,34)
Reime et al.^(24)^	324/8.857 (3,7)	124/8.857 (1,4)	24/8.857 (0,3)	73/8.857 (0,82)
Santos et al.^(25)^	115/537 (21,4)	107/537 (19,9)	-	-
Santos et al.^(27)^	120/549 (21,9)	113/549 (20,6)	-	-
Khashan et al.^(28)^	749/11.142 (6,7)	679/11.142 (6,1)	-	-
Mukhopadhyay et al.^(29)^	97/350 (27,7)	137/350 (39,1)	18/350 (5,1)	36/350 (10,3)
Martins et al.^(33)^	115/537 (21,4)	107/537 (19,9)	-	-
Santos et al.^(34)^	82/542 (15,1)	62/542 (11,4)	-	-

Total	4.449/51.966 (8,6)	4.021/51.966 (7,7)	223/38.659 (0,6)	138/9574 (1,44)

PT: pré-termo; BPN: baixo peso ao nascer.

Dois estudos^(20,27)^ que avaliaram a necessidade de internação em unidade de terapia neonatal (UTI) demonstram que 18,4% (119/648) dos recém-nascidos de mães adolescentes foram transferidos para UTI neonatal. Apenas um estudo descreveu a prevalência de morte infantil nessa população,^(21)^ que foi de, aproximadamente, 9,6 por mil nascidos vivos.

## DISCUSSÃO

Nas últimas décadas, têm-se discutido muito a respeito da adolescência, com uma ênfase maior no que diz respeito à complexidade e às repercussões da gravidez nessa fase. A gravidez, entre as adolescentes, é considerada um problema de saúde pública, que deve ser observado de forma ampliada, de maneira a envolver a mãe adolescente e os problemas que a cercam.^(35)^

No entanto, considerar a gravidez nessa fase como um fator de risco para desfechos adversos é algo redutor, uma vez que o fenômeno ocorre numa variedade de transações e que a vulnerabilidade, tanto da mãe quanto do bebê, pode ser diminuída por meio de fatores protetores.^(36)^ Desse modo, pode-se evidenciar que nem toda gravidez na adolescência é de alto risco obstétrico.^(37)^

Dentre os fatores de risco relacionados a gravidez na adolescência destacaram-se a baixa escolaridade, a idade da primeira relação sexual inferior a 15 anos, a ausência de companheiro, a história materna de gravidez na adolescência e a falta de conhecimento e de acesso aos métodos anticoncepcionais.^(38)^ Acrescentam-se a estes o abandono escolar, a ausência de planos futuros, a baixa autoestima, o abuso de álcool e drogas, a falta de conhecimento a respeito da sexualidade e o uso inadequado de métodos contraceptivos.^(39)^

Esses fatores podem influenciar os eventos reprodutivos adversos referentes a mãe adolescente e devem ser levados em consideração pelos programas de saúde pública durante a elaboração de estratégias para a prevenção da gravidez na adolescência.^(38)^

Ressalta-se que a gravidez na adolescência gera sérias consequências para o binômio mãe/filho, como, por exemplo, o desamparo e abandono da criança; os problemas emocionais; o afastamento escolar; a perda do emprego ou redução das opções de crescer no mercado de trabalho; e a multiparidade em um curto período de tempo.^(40)^

Estudo^(26)^ evidenciou que a proporção de mães adolescentes diminuiu progressivamente durante o período de 1999 a 2005. No Brasil, dados do Ministério da Saúde descreveram uma prevalência de gestação na adolescência de 20%. Em 2011, dos 2.913.160 nascidos vivos, 560.889 (19,2%) foram de mães adolescentes, sendo que 27.786 tinham idade inferior a 15 anos.^(41)^

Os estudos nacionais aqui apresentados^(20,25-27,29-30,32-33)^ demonstraram prevalência significativamente maior do que a apresentada pelo Ministério da Saúde, provavelmente por tais estudos terem sido realizados em serviços de atendimento terciário com maior prevalência de gestação de alto risco: 26,4% (1.623/6.149) *versus* 19,2% (560.889/2.913.160), com p<0,0001.

Na literatura em geral, alguns autores têm demonstrado aumento nas intercorrências materno-fetais em todas as etapas do ciclo gestacional entre gestantes adolescentes.^(7,8,42,43)^ No presente estudo, observou-se que as complicações associadas à gravidez na adolescência mais recorrentes na literatura estiveram mais associadas ao recém-nascido que propriamente à genitora, com predominância de manuscritos que enfatizaram a prematuridade, o BPN e a mortalidade. A ocorrência de nascimentos prematuros, recém-nascidos de baixo peso ou muito baixo peso e mortalidade foi significantemente maior entre os filhos de mães adolescentes.^(20,21,23-25,27-29,33,34)^

Essas intercorrências podem ser correlacionadas com o baixo número de consultas de pré-natal, o início tardio de pré-natal, o pré-natal inadequado e outros fatores, como raça, estado civil, baixa escolaridade, tabagismo e pobreza. Santos et al.^(34)^ observaram relação do BPN com o peso pré-gestacional, índice de massa corporal pré-gestacional e ganho de peso gestacional.

A literatura complementar sugere que os ambientes socioeconômico e cultural em que a jovem mãe está inserida estão associados ao aumento da frequência de recém-nascidos de baixo peso e prematuros. Além disso, sabe-se que os cuidados pré-natais tendem a ser inadequados entre as mães adolescentes,^(44)^ o que traz à tona a importância das consultas do pré-natal para diminuição das complicações da gravidez na adolescência.

A gravidez na adolescência é uma das três variáveis reprodutivas associadas à maior mortalidade infantil, principalmente por estar relacionada a uma complexa interação de fatores determinantes. No estudo de Oliveira et al.,^(30)^ a presença de comorbidade materna aumentou o risco para óbito fetal e pós-neonatal. Deve-se destacar que a maioria dos óbitos pode ser evitáveis e que as principais falhas se encontram na qualidade da assistência ao pré-natal, ao parto e à assistência neonatal.^(45)^

Analisando as intercorrências maternas relacionadas à gravidez na adolescência, a presente revisão sistemática encontrou uma menor quantidade de trabalhos relacionados ao tema^(20,23,25,31)^ quando comparado aos dados das intercorrências fetais. Foram citados: a pré-eclampsia, a eclâmpsia, HELLP, o abortamento, a infecção urinária e a ruptura prematura das membranas ovulares, entre outros. De forma geral, os trabalhos avaliados confirmaram que a gestante adolescente estaria mais propícia ao aumento de intercorrências maternas que uma gestante adulta.

A presença de comorbidade no período gestacional como, por exemplo, hipertensão, infecção do trato urinário, corrimento vaginal patológico, é muito mais comum entre adolescentes do que em outras idades.^(46)^

Por outro lado, a prevalência de cesariana nesse grupo foi significativamente menor quando comparada à população adulta. Dados do Departamento de Informática do Sistema Único de Saúde (DATASUS) revelaram que, entre 2008 e 2011, a prevalência de cesárea foi de 50,7% na população geral, sendo 43% na população adulta.^(41)^

Com relação à presença de pré-eclâmpsia e eclâmpsia como complicações da gravidez na adolescência, os resultados foram inconclusivos, em virtude de dados divergentes dos artigos selecionados. Alguns estudos citaram ser significantemente maior entre os filhos de mães adolescentes.^(20,23)^ Entretanto, outros observaram menor associação em mães adolescentes.^(25,27)^

Dos artigos analisados, três trabalhos citaram o aborto como um risco na gravidez precoce, enfatizando a expressão de não desejar a criança, não levando a termo a gestação.^(25,27,31)^ Santos et al.^(27)^ verificaram um risco de 2,34 de uso de abortivo no início da gestação em adolescentes grávidas. Correia et al.^(31)^ verificaram que 26,7% das adolescentes realizaram o aborto, principalmente por medo da família.

Abreu,^(47)^ ao analisar especificamente a ocorrência de abortamento na população de puérperas, encontrou uma proporção de adolescente com antecedentes de abortamento de 54,7% e constatou que a maioria tinha idade superior a 16 anos (89,7%). Com relação à quantidade de abortamentos, o mesmo autor observou a ocorrência de, no máximo, dois episódios de abortamentos entre as adolescentes.

Sabe-se que as adolescentes que abortam são vítimas de falta de informação, da deficiência no atendimento médico, da solidão e da falta de comunicação na família.^(48)^ Além disso, o abortamento é o responsável pelo aumento na frequência de internações hospitalares, podendo resultar em complicações físicas, psicológicas e até o óbito materno.^(49,50)^ Segundo Granja et al., cerca de 22% dos óbitos maternos referentes a gestantes adolescentes tiveram como principais causas a hipertensão induzida pela gravidez, a sepse puerperal e o abortamento séptico, representando 75% do total das mortes.^(51)^

A infecção urinária foi citada por dois estudos da presente revisão sistemática.^(20,27)^ Estudo de Nili et al.^(20)^ mostrou que apenas 2% das adolescentes grávidas apresentaram infecção no trato urinário. No estudo de Santos et al.,^(27)^ a infecção urinária ocorreu em 17,1% das adolescentes. Assim como a presente revisão sistemática, estudo teve por objetivo estabelecer o perfil da gravidez na adolescência em população assistida pelo Sistema Único de Saúde (SUS) no município de Muriaé, na Zona da Mata Mineira, e verificou que a infecção urinária foi uma das intercorrências mais frequentes entre as puérperas adolescentes, ocorrendo em maior proporção nas adolescentes com mais de 16 anos.^(47)^

No que se refere às complicações do parto, a ocorrência de ruptura prematura de membranas foi descrita em três artigos.^(20,23,27)^ Estudo relatou frequência de 20,3% de ruptura prematura em adolescentes grávidas, sugerindo que deficiências nutricionais podem desempenhar um papel importante nessa intercorrência.^(20)^

Yazlle et al. relataram intercorrências obstétricas em 38,3% das adolescentes e, entre os diagnósticos mais frequentes, encontravam-se os problemas fetais ou da placenta, e problemas com a cavidade amniótica e membranas.^(52)^

Apesar do aumento de cobertura para essa população na Atenção Básica, observam-se lacunas nos programas de educação em saúde e de prevenção que estimulem o uso de preservativos e contraceptivos, além de inexistirem políticas públicas direcionadas às jovens gestantes.^(38)^ Esses fatores colaboram para falta de conhecimento da adolescente quanto aos métodos de prevenção e para o aparecimento de uma gravidez indesejada e de suas possíveis complicações. Nesse contexto, justifica-se a necessidade de prevenção e de controle das consequências de uma gravidez precoce.^(40)^

Assim, cabe aos profissionais de saúde aprimorar a escuta, fortalecer vínculos com o jovem, garantir acesso a informações e aos métodos contraceptivos, e promover ações coletivas que auxiliem os adolescentes a lidarem com sua sexualidade, desenvolvam o autocuidado, e também ampliem o acesso a atividades educativas e recreativas.^(53)^

É importante ressaltar que esse estudo apresenta algumas limitações. Foram utilizadas apenas as bases de dados MEDLINE (PubMed), LILACS e SciELO, consideradas as principais na área de saúde. Entretanto, outras bases de dados poderiam ter sido consultadas, como EMBASE: *Biomedical Answers*, EBSCO e SCOPUS. Além disso, estudos transversais selecionados são difíceis de estabelecer com precisão a relação causa-efeito, pois a relação causal pode sofrer influências de fatores de confusão. A escassez de dados provenientes de ensaios clínicos controlados randomizados de qualidade para avaliar as complicações da gravidez na adolescência também foi um fator limitante.

## CONCLUSÕES

As principais complicações neonatais encontradas foram a prematuridade, o baixo ou muito baixo peso ao nascer e a mortalidade perinatal. Sugerem-se como principais complicações maternas a doença hipertensiva específica da gestação, o abortamento, a infecção urinária e a ruptura prematura das membranas ovulares. Entretanto, cabe enfatizar que os dados são controversos com relação à ocorrência de pré-eclâmpsia.

Nesse contexto, destaca-se a importância da realização de estudos para mais esclarecimentos sobre a mortalidade neonatal, que parece ser fortemente influenciada por determinantes como o baixo peso ao nascer e a prematuridade, e também sobre as intercorrências maternas relacionadas à gravidez na adolescência. Esse fato reforça a importância da prevenção dessas variáveis na assistência pré-natal e no parto.
